# Hydroxy-Group Topology as a Molecular Trigger Between Antioxidant and Photosensitizing Properties in Dihydroxynaphthalenes

**DOI:** 10.1021/acsomega.6c02192

**Published:** 2026-05-28

**Authors:** Plinio Innocenzi, Vasilis Petropoulos, Giulio Cerullo, Federico Olia, Davide Carboni, Luca Malfatti

**Affiliations:** † Laboratory of Materials Science and Nanotechnology, CR-INSTM, Department of Engineering, University of Sassari, Via Vienna 2, 07100 Sassari, Italy; ‡ Department of Physics, 18981 Politecnico di Milano, Piazza Leonardo da Vinci, 32, 20133 Milan, Italy

## Abstract

Dihydroxynaphthalenes (DHN) provide a simple molecular platform to probe how hydroxy-group topology governs the interplay between antioxidant activity and photoinduced reactive oxygen species generation. Four regioisomeric dihydroxynaphthalenes (1,3-, 1,5-, 1,8-, and 2,7-DHN) have been investigated by combining steady-state spectroscopy, time-correlated single-photon counting, and femtosecond transient absorption with functional assays. The results show that intramolecular hydrogen bonding and π-electron delocalization control excited-state relaxation pathways, determining the balance between nonradiative energy dissipation and intersystem crossing. Peri-hydrogen-bonded 1,8-DHN exhibits the highest radical-scavenging efficiency while suppressing triplet formation and singlet-oxygen generation, whereas isomers lacking intramolecular hydrogen bonding display enhanced photosensitizing behavior. These findings establish hydroxy-group topology as a key structural parameter linking ground-state redox chemistry with excited-state photophysics.

## Graphical abstract

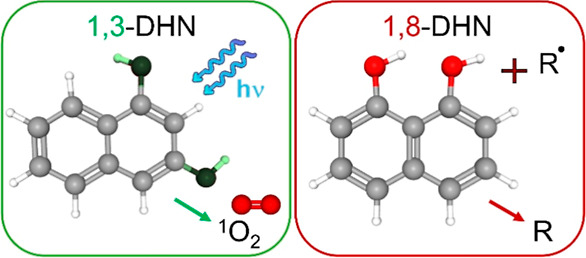

## 1 Introduction

Hydroxylated aromatic systems are fundamental structural elements in both natural antioxidants and synthetic chromophores, with the position and mutual interaction of hydroxy groups exerting a critical influence on their optical and redox properties.^1,2^

Dihydroxynaphthalenes (DHNs) provide a particularly suitable molecular platform to probe these effects, as different regioisomers exhibit distinct electronic coupling and hydrogen-bonding patterns already at the single-molecule level.^3^ These molecular-level differences can propagate across hierarchical length scales, ultimately shaping the redox and photochemical behavior of poly-DHN architectures such as allomelanins.^4–6^

In this work, we focus on the 1,3-, 1,5-, 1,8-, and 2,7-DHN isomers, which share the same molecular formula (C_10_H_8_) but differ in the position of the –OH functional group on the naphthalene parent structure formed by two fused benzene rings (Figure 1). The four isomers were selected because they form a minimal set that spans all the relevant hydroxy-group topologies on the naphthalene scaffold. Each isomer encodes a distinct pattern of π-electron delocalization and hydrogen-bonding capability: 1,8-DHN features a strong peri intramolecular O–H···O bond, 1,5- and 1,3-DHN allow only weaker or asymmetric intermolecular interactions, and 2,7-DHN represents a symmetric, non-H-bonded, para-like configuration. This choice enables a direct structure–property comparison, isolating how hydroxy topology alone controls the photophysical properties of the molecules.

**Figure 1:**
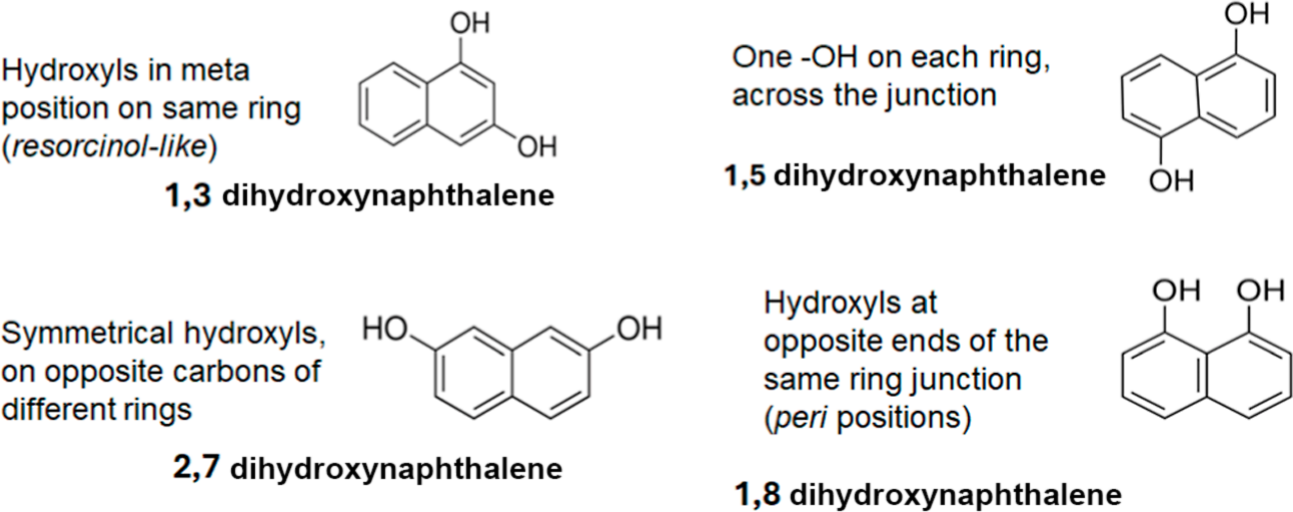
1,3-, 1,5-, 1,8-, and 2,7- dihydroxynaphthalenes. The different isomers are characterized by the position of the two hydroxys with respect to the parent structure formed by two bonded benzene rings.

The chemical and biological relevance of DHNs arises from their dual nature. On one hand, hydroxy groups can donate hydrogen atoms or electrons, stabilizing reactive radicals and conferring antioxidant properties similar to those of catechols (1,2-dihydroxybenzene) and phenolic compounds.^3,7–10^ On the other hand, when exposed to light, these same systems can populate excited triplet states and act as photosensitizers, generating reactive oxygen species (ROS), particularly singlet oxygen (^1^O_2_).^11^ Understanding how subtle structural variations control this balance between ROS generation and ROS quenching is crucial for developing photoactive antioxidants, photoprotective coatings, and environmentally benign sensitizers.^12,13^

Previous studies on polyhydroxylated aromatics have shown that intramolecular hydrogen bonding plays a decisive role in tuning electronic transitions and excited-state lifetimes.^14^ In peri-substituted systems such as 1,8-DHN, a strong O–H···O interaction can form a quasi-planar six-membered chelate, stabilizing the ground state and promoting nonradiative relaxation through excited-state proton transfer (ESPT) to the solvent. In contrast, meta- or para-substituted isomers like 2,7-DHN lack such internal coupling, leading to more symmetric electron distributions that can favor alternative excited-state relaxation pathways. Despite these theoretical insights, a comprehensive experimental comparison of dihydroxynaphthalene isomers, correlating their vibrational signatures, optical spectra, and photochemical reactivity, is still lacking.

In this work, we present a systematic study of the structure–property relationships in four DHN isomers, combining FTIR, UV–Vis absorption, photoluminescence, and time-resolved lifetime analyses with functional assays that probe their ability to scavenge free radicals (DPPH test) and generate singlet oxygen (ICG photobleaching assay). The spectroscopic results elucidate how hydrogen-bond topology modulates π-electron delocalization, while the reactivity tests reveal a tunable crossover between antioxidant and photosensitizing properties.

Through this integrated approach, we demonstrate that positional isomerism acts as a molecular switch controlling the optical gap, excited-state decay pathway, and redox function of dihydroxynaphthalenes, offering a rational basis for designing next-generation organic materials capable of tailored ROS control under light exposure.^15^

## 2 Experimental Section

### 2.1 Materials

1,3-Dihydroxynapthalene (1,3 DHN), 1,5-dihydroxynapthalene (1,5 DHN), 1,8-dihydroxynapthalene (1,8 DHN), 2,7-dihydroxynapthalene (2,7 DHN), 2,2-diphenyl-1-picrylhydrazyl (DPPH^·^) (C_18_H_12_N_5_O_6_), indocyanine green (ICG) (C_43_H_47_N_2_NaO_6_S_2_), and methanol (MeOH) (C_2_H_6_O, ≥99.9%) were purchased from Sigma-Aldrich and used without further purification.

### 2.2 Characterizations

Fourier transform infrared (FTIR) spectra were acquired in absorbance mode using a Vertex 70 interferometer in the range from 4000 to 400 cm^–1^ with 32 scans and 4 cm^–1^ resolution. The spectra were acquired from pellets of DHNs and potassium bromide (KBr) in a 1:200 weight ratio.

UV–vis spectra were collected using a UV–vis–NIR Cary 5000 spectrophotometer (Agilent) over a wavelength range of 200–900 nm with a bandwidth of 1.5 nm. Measurements were performed on 30 μM DHN solutions prepared in Milli-Q water, utilizing quartz cuvettes.

Three-dimensional fluorescence maps (*x*: emission; *y*: excitation; *z*: false color intensity) were acquired using a Horiba Jobin Yvon NanoLog spectrofluorometer equipped with a 450 W xenon lamp as the excitation source. Measurements were performed on 30 μM DHN solutions in Milli-Q water. Excitation–emission data were collected over an excitation range of 280–360 nm and an emission range of 280–500 nm, with 1 nm slits and 2 nm wavelength increments. Fluorescence lifetime measurements were conducted on the same instrument, using a NanoLED-340 as the excitation source at a wavelength of 330 nm.

Femtosecond transient absorption (fs-TA) measurements were performed using an amplified Ti/sapphire laser system (Coherent Libra, 800 nm, 80 fs, 1 kHz), providing an overall time resolution of ∼ 100 fs and a maximum pump–probe delay of 1 ns.^16^ Briefly, probe pulses were generated as a white-light continuum (320–680 nm) by focusing the 800 nm beam into a rotating 1 mm CaF_2_ plate, while 266 nm pump pulses were produced via third-harmonic generation of the fundamental beam using two β-barium borate crystals. Pump and probe polarizations were set at the magic angle (54.7°). Measurements were carried out in a 1 mm quartz flow cuvette, with 5 mL of solution continuously circulated and initially purged with N_2_ to prevent photochemistry. The optical density at 266 nm was kept below 0.05 (<0.3 OD at the low energy band), and the excitation fluence was maintained below 80 μJ cm^–2^ to minimize nonlinear effects. Data were analyzed by global fitting with multiexponential models using the Glotaran software.^17^

### 2.3 DPPH Radical Scavenging Assay

The radical scavenging activity was evaluated using the 2,2-diphenyl-1-picrylhydrazyl (DPPH) assay. A 0.25 mM DPPH solution was prepared in methanol. For the assay, 100 μL of the DPPH solution were mixed with 100 μL of different concentrations of DHNs (3.12, 6.24, 12.48, 18.72, 24.96, 31.2, 62.4, 93.6, 124.8 μΜ) in a 96-well microplate. The mixtures were incubated for 1 h in the dark at 25 °C. After the incubation, the absorbance at 517 nm was measured using a plate reader.

For evaluating the radical scavenging activity, the following formula was applied

1 $$Radical\;scavenging\;activity\;\left(\%\right)=\left(A_{517}C-A_{517}S\right)/\left(A_{517}C\right)x100$$

where A_517_C is the absorbance at 517 nm of the control, and A_517_S is the absorbance at 517 nm of the DHN solution. The control was prepared by mixing a DPPH methanol solution and water in a 1:1 ratio. The blank was prepared by mixing methanol and water in a 1:1 ratio.

### 2.4 Singlet Oxygen Generation Assay

The ability to generate singlet oxygen ^1^O_2_ was evaluated through the Indocyanine Green (ICG) assay. Briefly, a water solution with a concentration of 6.45 μM of DHN and 6.45 μM of ICG was prepared and transferred to a 3 mL quartz test cuvette. The mixture was irradiated at 330 nm for 5 min using a NanoLog Horiba Jobin Yvon spectrofluorometer with a 450 W xenon lamp in Real-Time Control (RTC) mode, equipped with a 4 nm slit aperture. A UV–vis spectrum was acquired using a Nicolet Evolution 300 spectrophotometer (Thermo Fisher) across a wavelength range of 200–900 nm with a bandwidth of 1.5 nm, recorded every 5 min during irradiation for up to 45 min. Photoinduced degradation of ICG was assessed by plotting the normalized absorbance at 782 nm and tracking its decrease over time. The photostability of ICG alone was evaluated using the same procedure.

## 3 Results and Discussion

### 3.1 UV–Visible Spectroscopy

Figure 2a shows the UV–vis absorption spectra of the four dihydroxynaphthalenes (1,3-, 1,5-, 1,8- and 2,7-DHN). The maxima of the different absorption bands are listed in Table 1. The different DHNs share a conjugated π-system (naphthalene core) and phenolic–OH groups that can increase the electron density of the aromatic ring. All DHNs show a strong absorption in the UV region (200–250 nm) that originates from π → π* transitions of the naphthalene aromatic system. It corresponds to the excitation from the highest occupied π-orbital, delocalized over the fused rings, to the lowest unoccupied π* orbital. Unsubstituted naphthalene shows three characteristic π–π* absorption bands at 312 nm (^1^B_3u_ ← ^1^A_g_), 280 nm (^1^L_b_ ← ^1^A_g_), and 220 nm (^1^L_a_ ← ^1^A_g_), as originally assigned by Platt^18^ and Birks.^19^ These transitions define the reference framework for the substituted dihydroxynaphthalenes analyzed here. In DHNs the position of the UV band becomes highly sensitive to substituent effects, in particular to the hydroxy substitution pattern, ortho/meta/para relative to the ring junction. Electron-donating groups (–OH) raise the HOMO energy (π orbital), which reduces the HOMO–LUMO gap and can cause a slight red-shift toward longer wavelengths. The extent of conjugation between the two hydroxy groups and the ring determines whether this red-shift is significant or nearly negligible. The UV band is also sensitive to possible intramolecular hydrogen bonding, which slightly delocalizes electron density and red-shifts the transition.

**Figure 2:**
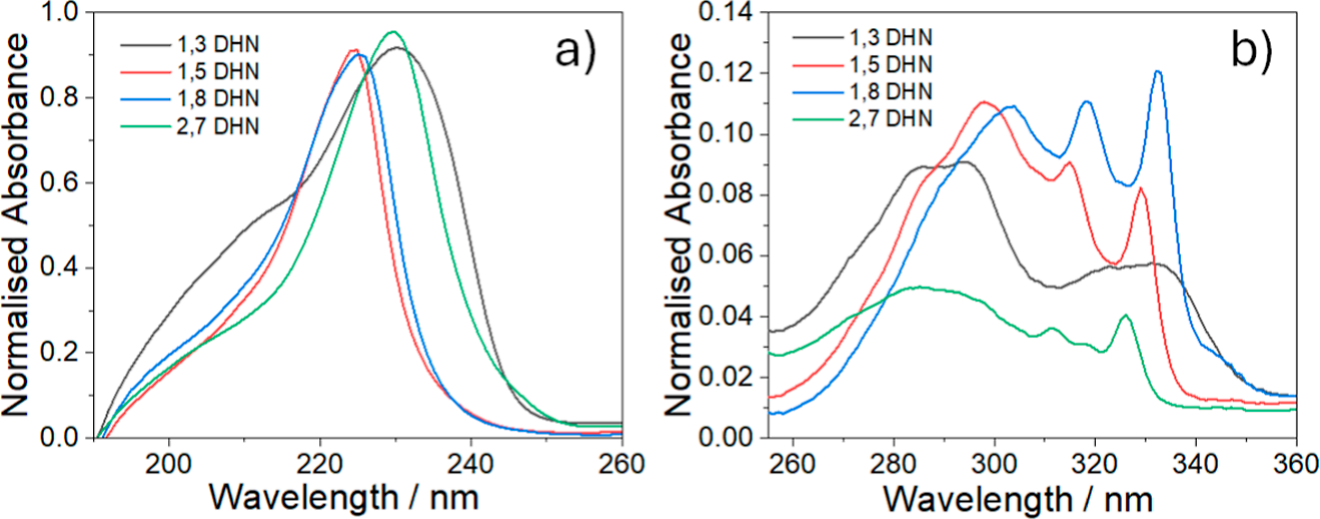
UV–visible absorption spectra of 1,3- (black line), 1,5- (red line), 1,8- (blue line), and 2,7-dihydroxynaphthalene (green line) in water (30 μM). The spectra show the high-energy ^1^L_a_ π → π* band near 200–260 nm (Figure 2a); a weaker ^1^B_b_ transition around 282–289 nm, and the structured ^1^L_b_ system (300–340 nm) (Figure 2b), whose position and vibronic detail depend on the hydroxy substitution pattern and intramolecular hydrogen bonding.

**Table 1:** DHN Maxima of the Different UV–Vis Absorption Bands Expressed in nm

molecule	deep UV	UV	I vibronic	II vibronic	III vibronic
**1,3 DHN**	230	285	295	322	333
**1,5 DHN**	224	286 (shoulder)	298	314	329
**1,8 DHN**	225	289 (shoulder)	303	318	331
**2,7 DHN**	229	282	311	319	326

The blue shift in 1,5-DHN (224 nm) reflects the reduced effective conjugation between the hydroxy substituents due to their relative positioning across the ring junction, which limits cooperative π-donation to the same aromatic subunit. The 1,3-DHN band at 230 nm, is red-shifted relative to 1,5-DHN (>6 nm) and likely arises from the higher polarizability of the resorcinol-type ring, which stabilizes the π → π* transition. 1,8-DHN maintains a comparable energy to 1,5-DHN but exhibits band broadening and a shoulder, consistent with dual contributions from localized and intramolecular H-bond-stabilized transitions. 2,7-DHN, being the most symmetrical and conjugated with hydroxys in para-like positions on opposite rings, shows a slight red shift in comparison to 1,5 and 1,8 DHN and greater intensity, indicating enhanced π-delocalization and lower transition energy.^20^

The deep UV band follows the sequence in increasing conjugation and red shift:

1,5DHN≈1,8DHN<2,7DHN≈1,3DHN

This progression reflects how substitution symmetry and inter-ring conjugation modulate the aromatic π-system. Symmetrical substitution (2,7-DHN) favors delocalization and a lower transition energy. These spectral differences serve as fingerprints of the electronic coupling between aromatic rings in the various dihydroxynaphthalene regioisomers.

The UV–vis region in Figure 2b (250–360 nm) displays several well-resolved absorption bands corresponding to vibronic structure arising from the coupling between electronic and vibrational states of the aromatic π-system in the dihydroxynaphthalenes (DHNs). When hydroxy groups are added (as in DHNs), the π-framework and the coupling with these vibrations are modified.^21^ The DHN patterns show a peculiar trend. 2,7-DHN shows a clear, three-band structure at 311, 319, 326 nm indicating a well-preserved vibronic progression, typical of a fairly rigid, symmetric naphthalene skeleton. In 1,5-DHN, the bands at 298, 314, 329 nm are broader, with less regular spacing because the substitution breaks the symmetry and slightly changes vibrational coupling. In 1,8-DHN the bands 303, 318, 331 nm are still visible but further broadened, due to intramolecular H-bonding, which distorts planarity and introduces anharmonic coupling. The bands 295, 322, 333 nm in 1,3-DHN are markedly broadened and less resolved because the resorcinol-like substitution localizes electrons on one ring and disrupts vibrational coherence. Each resolved band corresponds to transitions involving different vibrational quanta (v′) of the same electronic excited state. The first band (lowest energy, longest wavelength) → 0–0 transition (no change in vibration). The subsequent bands → 0–1, 0–2, etc., involving excitation of one or more quanta of the aromatic C=C stretch or ring-breathing modes. The approximate energy spacing (Δ*E*) between absorption peaks (≈800–1200 cm^–1^) matches typical aromatic stretching frequencies, confirming their vibronic origin.

The hydroxy substituents in DHNs alter vibronic coupling by changing mass distribution and force constants of the ring. They provide electron donation through resonance, modifying π orbital symmetry and forming intra- or intermolecular hydrogen bonds, introducing new low-frequency modes (O–H···O stretching, bending). Another effect is the reduction of planarity or symmetry, which leads to broader, less regular vibronic patterns. Therefore, symmetric, rigid systems (2,7-DHN) give a well-resolved vibronic structure while asymmetric or H-bonded systems (1,8- and 1,3-DHN) produce broadened, merged vibronic bands.

Between the UV and vibronic bands another intermediate absorption band is observed around 282–289 nm. This ≈ 280–290 nm band appears between the intense π → π* transition near 220–230 nm (the ^1^L_a_-type) and the structured long-wavelength system around 300–330 nm (the ^1^L_b_-type). It represents excitation from a deeper π orbital (often HOMO–1) to an antibonding π*, involving orbitals with different nodal symmetry than in the ^1^L_b_ transition. This intermediate band serves as a bridge between the two major π → π* systems, it represents delocalized transitions across the naphthalene framework. The band may mix with the high-frequency vibronic components of the ^1^L_b_ system. It provides additional information on how the electronic density is redistributed by –OH substitution. In practice, the 2,7-DHN 282 nm band is distinct, reinforcing its rigid, naphthalene-like nature. The 1,5- and 1,3-DHN with the 285–286 nm shoulder merges with the onset of the long-wavelength ^1^L_b_ band. The 1,8-DHN 289 nm feature broadens, as H-bonding increases vibronic coupling and internal relaxation. The and at 282–289 nm in dihydroxynaphthalenes corresponds to a ^1^B_b_-type π → π* transition, an intermediate excitation between the ^1^L_a_ and ^1^L_b_ systems. The different transitions observed in DHNs are resumed in Table 2.

**Table 2:** Conceptual Summary of the Different Transitions in the DHN UV–Vis Absorption Spectra

transition	typical range (nm)	nature	sensitivity
^**1**^ **L**_**a**_	220–230	intense, long-axis	modestly affected by substitution
^**1**^ **B**_**b**_	∼280–290	intermediate π → π*	strongly affected by conjugation and H-bonding
^**1**^ **L**_**b**_	300–340	low-energy π → π*, vibronically structured	highly sensitive to symmetry, planarity

### 3.2 Photoluminescence

Figure 3 shows the photoluminescence 3D spectra of DHNs. The fluorescence of the four DHN isomers reflects the strong influence of hydroxy substitution on the π-electron delocalization evidenced by their UV–vis spectra (Figure 2). All isomers exhibit their main emission peaks in the near-UV region upon excitation within the long-wavelength ^1^L_b_ absorption band (≈300–340 nm), confirming that their dominant fluorescent channel arises from the lowest singlet excited state (S_1_) derived from the ^1^L_b_ π → π* manifold after rapid internal conversion from higher states.

**Figure 3:**
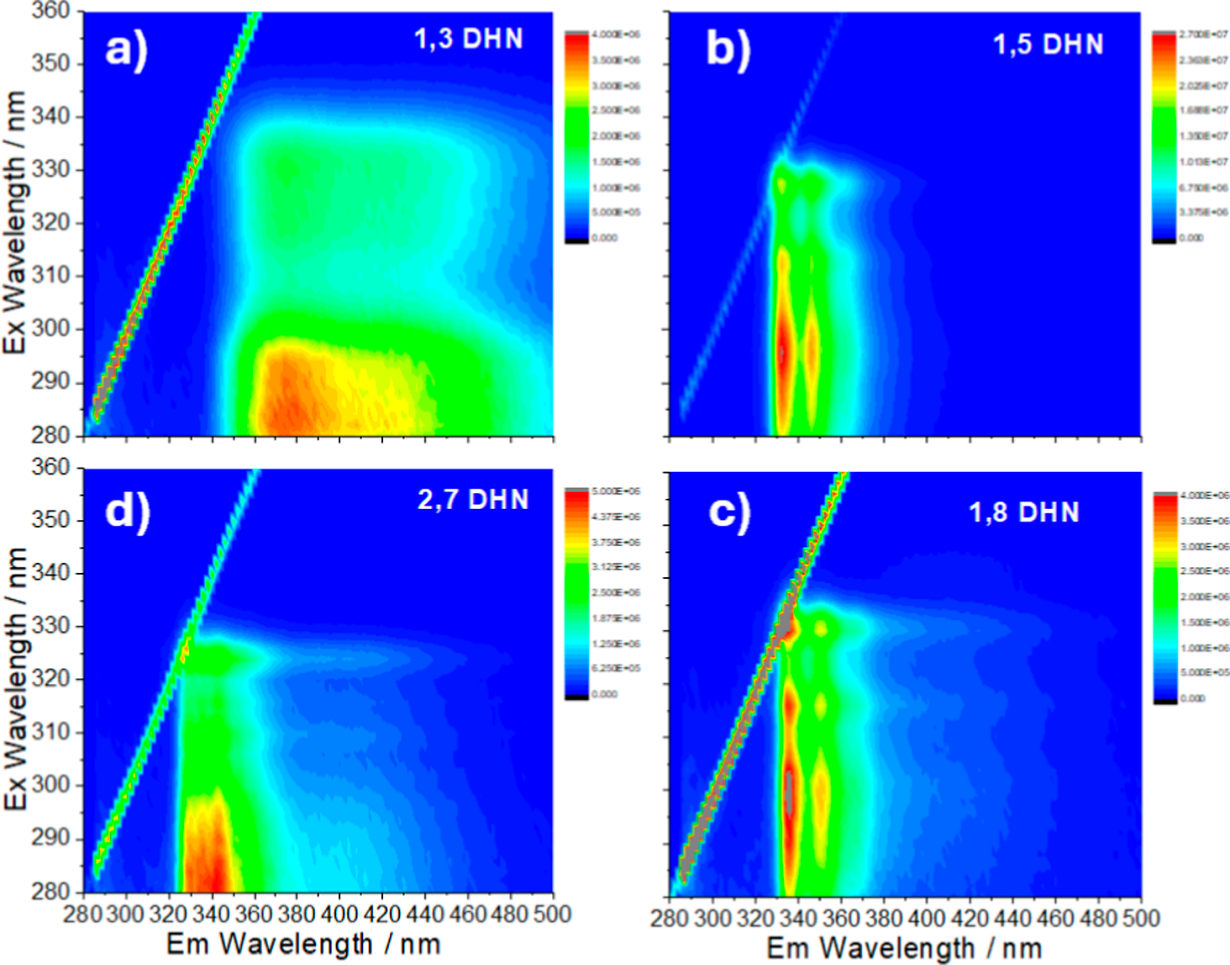
Photoluminescence 3D spectra of DHNs (30 μM solution in milli-Q water); (*x*-emission wavelength; *y*-excitation wavelength; *z*-false colors intensity scale). (a) 1,3 DHN; (b) 1,5 DHN; (c) 1,8 DHN; (d) 2,7 DHN.

1,3-DHN shows two large emissions that extend from 340 up to 500 nm. The emissions are activated by excitations in the 280–305 and 305–340 nm ranges, respectively, peaking around 285 and 330 nm. In 1,3-DHN the two hydroxys are in a “resorcinol-like” meta-arrangement on the same ring system, which localizes electron density and breaks symmetry. This behavior is already evident in the UV–vis spectra, where the low-energy vibronic structure (∼295, 322, 333 nm) appears broadened and poorly resolved, indicating increased vibrational dephasing due to localized electronic character.

1,5 and 1,8 DHNs show, instead, a quite different emission with respect to 1,3-DHN; the emissions are much narrower and in the 330–370 nm range. They exhibit six different emission centers peaking at 332 (λ_ex_ = 313 nm), 332 (λ_ex_ = 296 nm), 332 (λ_ex_ = 327 nm), 346 (λ_ex_ = 296 nm), 346 (λ_ex_ = 313 nm), 346 (λ_ex_ = 326 nm) in the 1,5-DHN sample and at 335 (λ_ex_ = 299 nm), 335 (λ_ex_ = 315 nm), 335 (λ_ex_ = 330 nm), 350 (λ_ex_ = 299 nm), 349 (λ_ex_ = 315 nm), 349 (λ_ex_ = 330 nm) in the 1,8-DHN sample.

Both 1,5-dihydroxynaphthalene and 1,8-dihydroxynaphthalene display remarkably similar UV–vis absorption envelopes and near-UV fluorescence bands, with only minor shifts in peak position and bandwidth. This indicates that, in aqueous environment, both isomers relax toward electronically comparable S_1_ emissive states that are already partially vibrationally cooled and largely structureless, in contrast to the more vibronically resolved behavior of 2,7-DHN and the strongly red-shifted, broader emission of 1,3-DHN.

On the other hand, 2,7-DHN emission range is similar to 1,5 and 1,8 DHN but with only two clear emission centers, peaking around 285–340 nm and 325–340 nm. 2,7-DHN is the most symmetric isomer, with hydroxy groups located on opposite rings in positions that preserve overall molecular symmetry.

The fluorescence map of 2,7-DHN also shows relatively structured emission, with clear emission centers and vibronic spacing of roughly 13–15 nm. That spacing is typical of aromatic C=C stretching modes and indicates that the excited state geometry is close to the ground state geometry, i.e. it shows little structural relaxation. As a result, the Stokes shift is moderate, and the emission is comparatively efficient.

### 3.3 FTIR Structural Analysis

The FTIR spectra of the four dihydroxynaphthalene (DHN)^22^ isomers exhibit well-defined O–H and C=C stretching bands, whose positions and shapes are diagnostic of intramolecular hydrogen bonding and aromatic conjugation (Figure 4).

**Figure 4:**
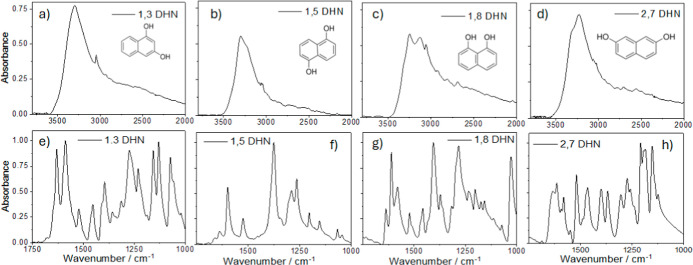
FTIR absorption spectra in the 3700–2000 cm^–1^ range (a–d) and 1750–1000 cm^–1^ range (e–h).

All infrared spectra show a broad O–H stretching region between 3600–3200 cm^–1^, but the profile varies significantly among isomers (Figure 4a–d). A closer look at the OH stretching band shows some interesting peculiarities that can be used to clarify the differences in H-bonding environments between DHNs. 1,3-DHN shows a single band peaking at 3314 cm^–1^. The meta position of OH in 1,3 DHN topology does not favor a tight intramolecular chelate.

1,5-DHN, instead, shows a main OH band peaking at 3289 cm^–1^ and an overlapped band at 3217 cm^–1^ (shoulder). This indicates the presence of two O–H subpopulations. The 3217 cm^–1^ is due to stronger H-bonded OH (tighter intermolecular links/shorter O···O), while the 3289 cm^–1^ to weaker H-bonded OH (looser contacts or less favorable geometry). The 1,5 arrangement supports asymmetric intermolecular networks (chains/dimers) where one OH often acts as the better donor/acceptor than the other.

18-DHN shows two distinct bands peaking at 3241 cm^–1^ and 3121 cm^–1^. The two bands can be assigned to clear intramolecular *peri* O–H···O H-bonding that creates strong inequivalence. The 3121 cm^–1^ band is very strongly H-bonded OH (dominant donor within the intramolecular chelate) and appears red-shifted. The 3241 cm^–1^ is the less strongly H-bonded partner (or an OH sampling weaker inter/intramolecular contacts). The *peri* geometry (1 and 8) supports a tight intramolecular H-bond, giving one OH a much softer (more anharmonic) potential with a consequent large downshift and distinct splitting.

2,7-DHN spectra exhibit one main band 3255 cm^–1^ and a second one at 3311 cm^–1^ (shoulder). This is indicative of a mixed OH population. The 3311 cm^–1^ component is due the weakly H-bonded/quasi-free OH fraction, while the 3255 cm^–1^ band to a moderately H-bonded OH fraction (intermolecular). This pattern is due to the para-like separation that precludes intramolecular H-bonding. It is, therefore, observed the coexistence of loosely associated and more engaged intermolecular sites with a higher-frequency shoulder plus a main band.

The spectra show also an overlapped sharp peak at 3055 cm^–1^. It is the aromatic C–H stretching band (sp^2^ ν­(CH)) that sits at the high end of the C–H region (≈3100–3000 cm^–1^) and happens to overlap the low-frequency tail of the broad H-bonded O–H envelope. It is detected, with small changes, in all four isomers, because it tracks the naphthalene ring rather than the OH topology.

Interestingly, the infrared spectra show an overlapped faint absorption band around 2500 cm^–1^ that form a long tail into the lower wavenumber region. This wide band does not correspond to any fundamental stretching vibration of the DHN skeleton itself. It is, in fact, a combination/overtone band, and its appearance is diagnostic of strong hydrogen bonding involving O–H groups. The ≈2500 cm^–1^ band is attributed to Fermi resonance between the ν­(OH) stretching mode and overtone/combination bands of O–H bending vibrations, consistent with strongly hydrogen-bonded OH groups.

Figure 5 shows the infrared aromatic ring–stretch zone in the 1700–1500 cm^–1^ window. In this range, ν­(C=C) ring (8*a*/8b pair) is typically observed around ∼1640–1600 (C=C stretch along the long molecular axis (*a*-axis)) and ∼1600–1580 cm^–1^ (C=C stretch along the short molecular axis (*b*-axis)). Both are aromatic ring-stretching vibrations, largely localized on the conjugated C=C framework, and their frequency difference (Δν ≈ 30–40 cm^–1^) reflects how substituents or hydrogen bonding perturb the π-electron density along different molecular directions (usually red-shift with stronger O–H···O coupling). In the ∼1530–1515 cm^–1^ range, the ring stretching is mixed with the in-plane C–H bending vibrations δ­(CH) (ν_19_ *a*/ν_19_ *b*). Substitution by hydroxy groups perturbs this manifold through both electronic donation to the π-system and hydrogen-bond interactions, producing systematic frequency shifts and band splittings that reflect the local environment of each ring.

**Figure 5:**
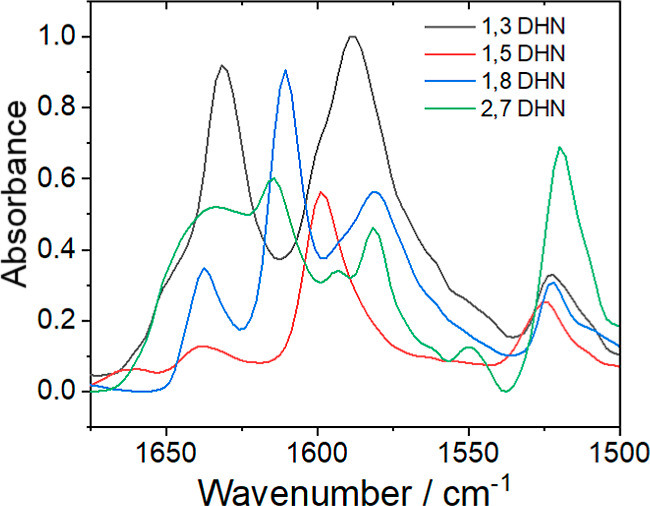
FTIR absorption spectra of 1,3-DHN (black line), 1,5-DHN (red line), 1,8-DHN (blue line), 2,7-DHN (green line) in the 1700–1500 cm^–1^ range. The bands correspond mainly to the ν­(8*a*) and ν­(8*b*) ring-stretching vibrations and the ν(19) in-plane C–H bending modes of the naphthalene framework. Progressive red-shifts and splittings of these modes reveal the influence of hydroxy substitution and hydrogen bonding: 1,8-DHN shows the strongest red-shift (to 1580 cm^–1^) and pronounced 8*a*/8*b* splitting due to an intramolecular *peri* O–H···O bond, 1,5-DHN exhibits moderate shifts with a minor 1683 cm^–1^ shoulder attributable to trace oxidation, 1,3-DHN retains nearly symmetric aromatic stretches, and 2,7-DHN displays the weakest perturbation and multiple narrow components typical of weak, intermolecularly H-bonded species.

The infrared spectra of 1,3-DHN show two bands 1631 (8*a*), 1587 cm^–1^ (8*b*) that are both near parent naphthalene values. In the 1,8-DHN the bands are detected at 1637 (8*a*), 1610 and 1580 cm^–1^ (split 8*a*/8*b* due to peri H-bond). 2,7-DHN shows multiple 8*a*/8*b*-derived components at 1634 (8*a*) + 1614, 1593, 1581 cm^–1^. These shifts and splittings trace how hydroxy substitution and hydrogen bonding distort the π-framework.

The 1,5-isomer shows bands at 1683, 1599, and 1524 cm^–1^; the weak feature at 1683 cm^–1^, absent in the other spectra, may arise from a trace of quinone-type oxidation or from an overtone combination, while the principal 8*a*/8*b* positions remain close to those of unsubstituted naphthalene. The 1,8-isomer exhibits four components at 1637, 1610, 1580, and 1521 cm^–1^. The pronounced splitting of the 8*a*/8b pair and the marked red-shift of the lowest band (1580 cm^–1^) indicate strong π-electron delocalization mediated by the intramolecular *peri* O–H···O hydrogen bond. Conversely, 2,7-dihydroxynaphthalene displays the richest structure (1634–1519 cm^–1^) but the smallest overall red-shifts, consistent with the absence of intramolecular coupling and with weak, predominantly intermolecular hydrogen bonding.

Overall, the gradual red-shift of the 8b component and the increasing 8*a*/8b splitting follow the sequence 2,7 < 1,3 ≈ 1,5 < 1,8, mirroring the growth of hydrogen-bond strength and π-conjugation along the series. These ring-stretching features, therefore, provide a sensitive vibrational fingerprint for evaluating the electronic consequences of hydroxy substitution and intramolecular hydrogen bonding in dihydroxynaphthalenes.

The FTIR measurements were performed in the solid state and are therefore not intended to quantify hydrogen-bond association energies or solution-phase equilibria. The vibrational signatures are rather used to identify the intrinsic hydrogen-bonding motifs encoded by hydroxy-group topology, in particular the unique peri O–H···O interaction in 1,8-DHN. These structural features provide a rational basis for interpreting the distinct photophysical and redox behaviors observed in solution.

It is worth noting that, although comparative measurements in non-hydrogen-bonding solvents can in principle help disentangle intra- and intermolecular hydrogen-bonding contributions, such experiments are not feasible in the present system due to the very limited solubility of dihydroxynaphthalenes in apolar, non-hydrogen-bonding media (e.g., CCl_4_). Attempts to perform spectroscopic measurements under these conditions did not yield homogeneous solutions at concentrations suitable for reliable analysis. Therefore, the identification of hydrogen-bonding motifs in this work relies on intrinsic vibrational signatures (solid-state FTIR) and on internally consistent trends observed in solution-phase optical and time-resolved spectroscopies. In particular, intramolecular hydrogen bonding, such as the peri O–H···O interaction in 1,8-DHN, is an intrinsic structural feature that can be inferred primarily from intrinsic structural and vibrational signatures, largely independent of solvent effects.

### 3.4 Excited-State Lifetime

The assignment of the excited-state manifolds discussed below (e.g., ππ* character, S_1_ minima, and relaxation pathways) is based on a combination of established literature on naphthalene and hydroxylated aromatic systems and on photophysical interpretation of the experimental observables (steady-state spectra, TCSPC lifetimes, and transient absorption data). No explicit quantum-chemical calculations have been performed; therefore, the proposed state assignments should be regarded as physically consistent models rather than direct electronic-structure determinations.

Figure 6 shows the decay times calculated for the four different DHNs. 1,5, 1,3, and 2,7 DHNs exhibit only one decay time in the ns scale, τ_1_ = 8.2 ± 0.1, 7.4 ± 0.1 and 5.8 ± 0.1 ns, respectively. 1,8 DHN decay can be simulated, instead, using two decay times, one faster: τ_1_ = 2.5 ± 0.1 ns and another slower τ_2_ = 11.7 ± 1.0 ns.

**Figure 6:**
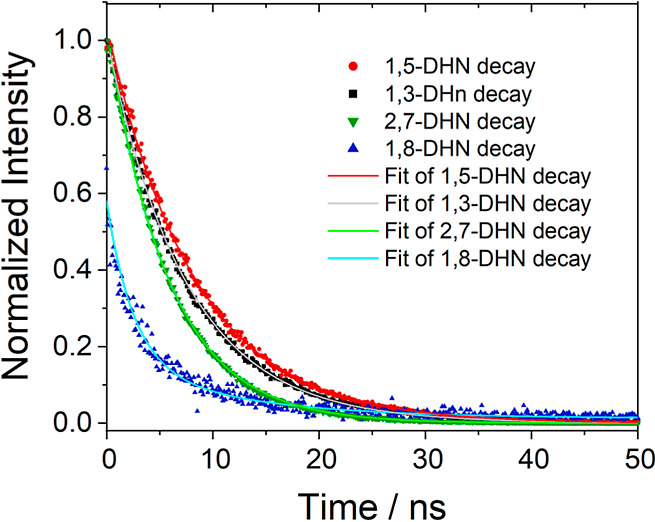
Decay times calculated for the different DHNs. 1,5 (red line), 1,3 (black line), 2,7 (green line) and DHNs exhibit only one decay time in the ns scale, τ_1_ = 8.2 ± 0.1, 7.4 ± 0.1 and 5.8 ± 0.1, respectively. 1,8-DHN (blue line) decay can be simulated, instead, using two decay times, one faster: τ_1_ = 2.5 ± 0.1 ns, and another slower, τ_2_ = 11.74 ± 1.0 ns. The symbols show the experimental points and the continuous lines the fits.

The fluorescence lifetime data (Figure 6 and Table 3) reveal that subtle variations in hydrogen-bond topology profoundly affect excited-state relaxation in the DHN isomers. The single-exponential decays observed for 1,3-, 1,5- and 2,7-DHN, indicate that the emissive singlet state (S_1_ → S_0_) dominates their photophysics, with limited contribution from alternative emissive channels. The decay time change, 1,5 → 1,3 → 2,7, reflects differences in excited-state relaxation efficiency associated with molecular rigidity and electronic symmetry.

**Table 3:** Fluorescence Lifetimes of Dihydroxynaphthalene (DHN) Isomers Measured by TCSPC

molecule	τ_1_ (ns)	τ_2_ (ns)	decay Model	assignment
1,3-DHN	7.4 ± 0.1	—	single exponential	S_1_ → S_0_ radiative decay
1,5-DHN	8.2 ± 0.1	—	single exponential	S_1_ → S_0_ radiative decay
2,7-DHN	5.8 ± 0.1	—	single exponential	S_1_ → S_0_ radiative decay (rigid π-system)
1,8-DHN	2.5 ± 0.1	11.7 ± 1.0	biexponential	dual pathway: ESIPT/H-bond relaxed states

By contrast, the biexponential behavior of 1,8-DHN (τ_1_ = 2.5 ns, τ_2_ = 11.7 ns) evidence two emissive species, consistent with dual relaxation pathways. The shorter component likely corresponds to an excited-state intramolecular proton-transfer (ESIPT) process within the strong peri O–H···O bond, while the longer one reflects radiative decay from a stabilized tautomeric or H-bond-relaxed form. This coexistence of fast and slow channels indicates that intramolecular hydrogen bonding not only stabilizes the ground state but also introduces additional relaxation coordinates that modulate the lifetime and overall quantum yield.

To elucidate the early time relaxation pathways that precede fluorescence, femtosecond transient absorption (fs-TA) spectroscopy was performed on the four DHN isomers in aqueous solution. Samples were excited with ∼100 fs pulses at 266 nm (see Experimental Section), populating the ^1^B_b_-type π–π* excited-state manifold of the dihydroxynaphthalene chromophores.

Figure 7 (upper panels) shows the TA maps of 1,3-DHN, 1,5-DHN, 1,8-DHN, and 2,7-DHN (from left to right) as a function of probe wavelength and pump–probe delay.

**Figure 7:**
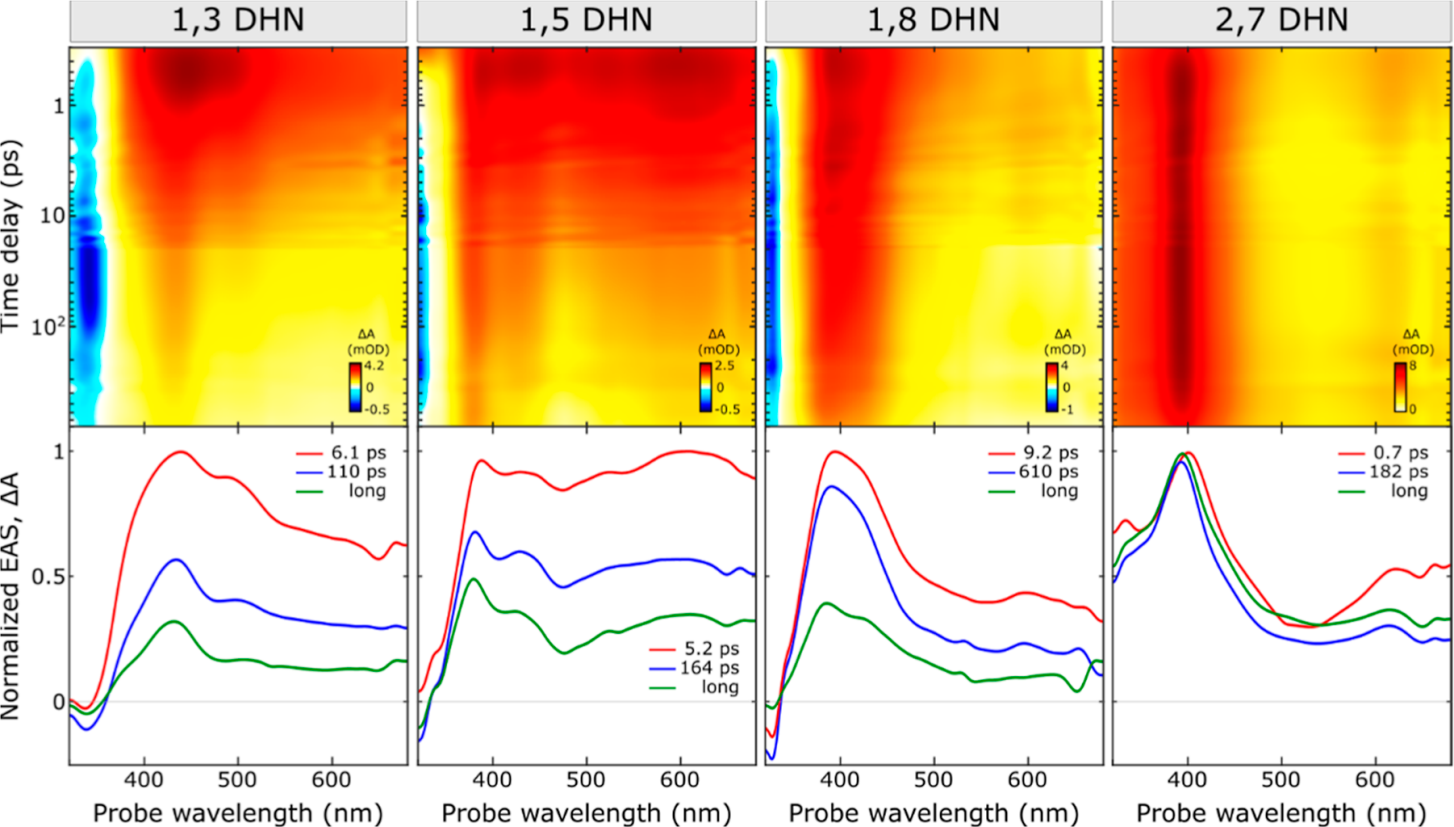
Transient absorption (TA) maps (upper panels) of the four DHN isomers studied (1,3-DHN, 1,5-DHN, 1,8-DHN, and 2,7-DHN; from left to right) are shown as a function of probe wavelength and pump–probe delay following excitation at 266 nm. The corresponding evolution-associated spectra (EAS) obtained from global analysis of the TA data are displayed in the lower panels, yielding three EAS components. TA maps are presented on a logarithmic time scale and shown as the measured differential absorbance (Δ*A*). The first EAS component is normalized, while the relative amplitudes of the subsequent components are preserved for comparison.

Immediately after excitation, all isomers exhibit a broad photoinduced absorption (PA) extending across the visible region, partially overlapping negative features below ∼400 nm attributed to ground-state bleaching (GSB) and, where present, stimulated emission (SE). For 1,3-DHN, the PA spans approximately 350–680 nm with dominant intensity in the 420–500 nm region and a weaker long-wavelength contribution near ∼620 nm. A similarly broad but less structured PA is observed for 1,5-DHN, featuring multiple peaks over the visible spectrum. In contrast, 1,8-DHN and 2,7-DHN display more structured PA, with a pronounced band in the near-UV/blue (∼390 nm) and a weaker band in the red region (∼620 nm). As the initial internal conversion from the populated ^1^B_b_-type state to the vibronically coupled ^1^L_b_ occurs typically faster than the experimental time resolution, this broad PA signal represents mainly the population of the hot S_1_ (^1^L_b_ in character) manifold with small contributions possibly coming from the higher electronic state.

Within the first few picoseconds, the initially broadband PA evolves into a more structured spectral pattern for all isomers, accompanied by a relative blue shift of the PA, as reflected by an ∼5 nm shift of its peak and by the increasing prominence of the 400–450 nm region relative to the 600–650 nm region. This evolution is accompanied by growth or unmasking of the negative band below ∼400 nm for all isomers except 2,7-DHN, which exhibits predominantly positive signals of high oscillator strength in the probed range, masking the GSB/SE contribution. This early time evolution is assigned to ultrafast relaxation within the S_1_ excited-state manifold, including vibrational cooling and solvent- and hydrogen-bond reorganization leading to formation of a relaxed S_1_ configuration.

Following this initial relaxation, an isosbestic point becomes evident near ∼340–370 nm for 1,3-DHN, 1,5-DHN, and 1,8-DHN, indicating kinetic interconversion of a well-defined relaxed emissive excited state into a product population. In this time regime, decay of the PA is accompanied by partial GSB recovery together with the persistence of a long-lived excited population. This behavior is consistent with irreversible branching of the relaxed S_1_ population on the subnanosecond time scale, rather than complete S_1_ → S_0_ relaxation within the experimental time window.

To quantify these dynamics, the TA data sets were analyzed by global fitting using a sequential three-component model (Figures S1–S4). The resulting evolution-associated spectra (EAS; Figure 7, lower panels) reveal a common relaxation scheme across all isomers, modulated by hydroxy-group topology. The first fitting component (τ_1_ ≈ 0.7–9 ps) describes relaxation from an admixture of vibrationally hot S_1_ states to a relaxed emissive geometry, reflected by spectral structuring of the PA and growth of the GSB/SE region. Notably, the most rigid isomer, 2,7-DHN, exhibits the fastest τ_1_ (∼0.7 ps), while 1,8-DHN shows the slowest relaxation (∼9 ps), consistent with the need for additional nuclear and solvent reorganization within the peri hydrogen-bonded pocket. Overall, τ_1_ follows the trend 2,7-DHN < 1,5-DHN ≈ 1,3-DHN < 1,8-DHN.

The second component (τ_2_ ≈ 100–600 ps) corresponds to subnanosecond decay of the relaxed S_1_ spectral signature around the isosbestic point and is assigned to branching of the relaxed excited-state population into partial ground-state recovery and formation of long-lived excited configuration(s). The third component (τ_3_ ≫ 1 ns) accounts for decay of the residual excited population persisting beyond the 1 ns delay range and is consistent with the nanosecond fluorescence lifetimes observed by time-correlated single photon counting (TCSPC).

The τ_2_ lifetime that precedes fluorescence and describes branching of S_1_ to other states, can be greatly affected by the number of hydroxy groups and their topology. For instance, in monohydroxylated naphthalene (1-naphthol), excited-state proton transfer (ESPT) to water occurs within ∼ 33 ps, leading to dual emissive species and a dominant red-shifted anionic emission in steady-state spectra.^23^ In contrast, in structurally similar dihydroxy aromatic systems, ESPT, if present, is considerably slower and competes with fluorescence and triplet formation rather than dominating the excited-state relaxation.^24,25^ It should be noted, however, that the formation of long-lived dark states does not necessarily imply efficient triplet sensitization, as their population and oxygen-coupling efficiency strongly depend on molecular symmetry and spin–orbit coupling.

Indeed, the predominance of vibronically structured steady-state emission (see Figure 3), mirror-imaged to the absorption spectra, together with largely single-exponential TCSPC decays indicates that fluorescence originates mainly from a single neutral emissive ensemble on S_1_ for 1,5-DHN and 2,7-DHN. For 1,8-DHN, although the fluorescence remains vibronically structured and largely originates from S_1_, the presence of two lifetimes in TCSPC is consistent with water-gated slow ESPT occurring along the τ_2_ time scale and possibly followed by proton redistribution within the peri hydrogen bond.^4^

On the other hand, 1,3-DHN is the only isomer exhibiting dual emission (see Figure 3), similar to 1-naphthol; however, in this case the neutral S_1_ emission remains predominant. As a result, any ESPT channel is likely embedded within the τ_2_ component, potentially shortening its lifetime without producing a clearly resolved second SE band in the TA spectra, possibly due to the low quantum yield and oscillator strength of the anionic species formed. The single-exponential TCSPC decay observed in this case may therefore reflect two emissive species with similar lifetimes.

A recent study by Lino et al. on dihydroxynaphthalenes^3^ established 1,8-DHN as the most efficient antioxidant under dark conditions. The antioxidant stability has been attributed to aryloxyl radical stabilization by peri intramolecular hydrogen bonding and favorable hydrogen-atom transfer kinetics. Our results fully confirm this antioxidant hierarchy but extend the underlying structure–function relationship into the excited-state regime. Time-resolved fluorescence and femtosecond transient absorption reveal that the same peri O–H···O interaction in 1,8-DHN promotes efficient nonradiative dissipation and suppresses triplet formation, thereby limiting singlet-oxygen generation. In contrast, isomers lacking intramolecular hydrogen bonding display enhanced intersystem crossing and higher photosensitizing activity, identifying hydroxy topology as a key trigger for antioxidant and photoactive properties.

In summary, while monohydroxylated naphthalene behaves as an ideal photoacid with efficient formation of a bright anionic excited state, the introduction of a second hydroxy group creates a frustrated hydrogen-bonding and electronic landscape that favors excited-state branching and long-lived neutral emission over clean ESPT. Furthermore, the relative topology of the two hydroxy groups in the DHN isomers can further tune the branching of the relaxed S_1_ population between the ground state and other excited-state pathways.

### 3.5 Pro-Oxidant/Radical Scavenging Properties

To measure the different capability of the isomers to act as radical scavengers a DPPH assay has been used (Figure 8a). It is important to stress that DPPH is used here as a comparative probe for radical scavenging and does not provide direct kinetic information on antioxidant efficacy in the strict sense, which is formally defined by the rate constants of reactions with peroxyl radicals. Consequently, the presented results reflect the relative radical scavenging activity and stoichiometry under fixed experimental conditions, rather than the intrinsic rate constants of hydrogen atom transfer. 1,8 DHN is the most efficient isomer as radical scavenger and its activity quickly rises to 100% at the concentration around 20 μM. 1,5 DHN also reaches a 100% of activity but at a higher concentration (70 μM). The other two isomers, 2,7 and 1,3 DHN reaches a scavenger activity of around 80% but only at much higher concentrations in comparison to 1,8 and 1,5 DHN.

**Figure 8:**
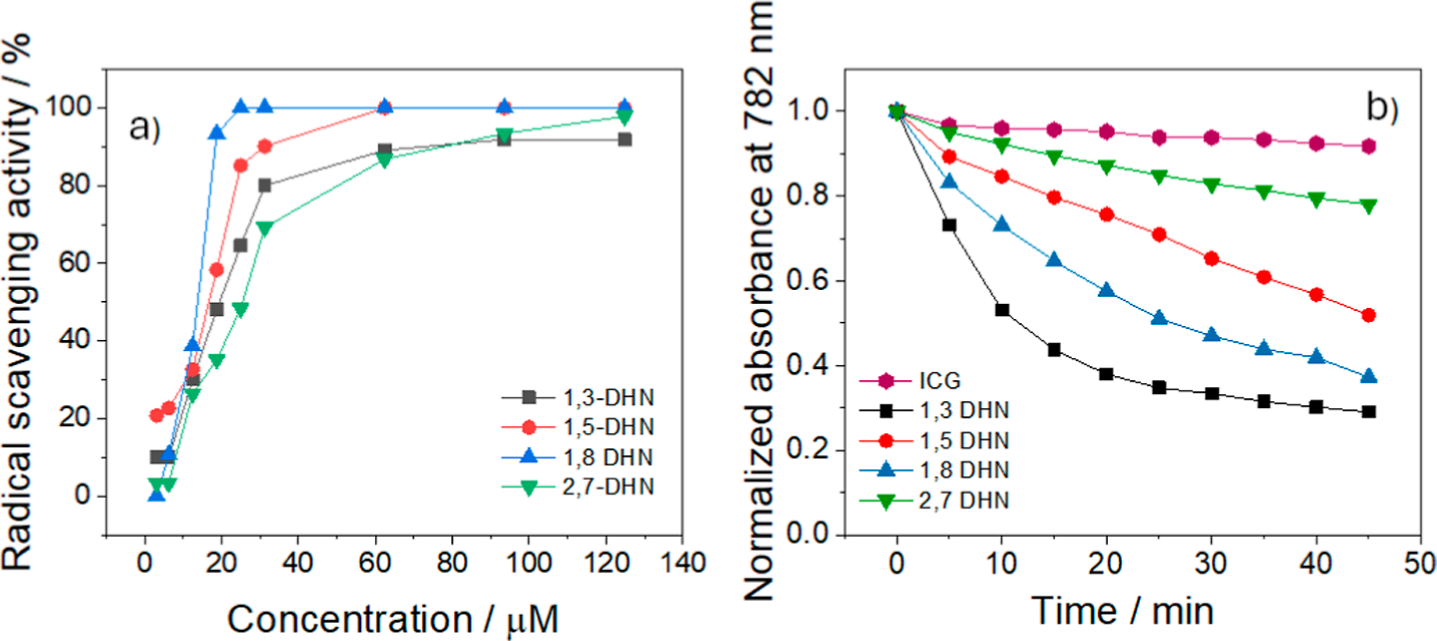
(a) DPPH assay to test the radical scavenging activity of the different DHN isomers. (b) Isocyanine green assay to measure the capability of generating singlet oxygen upon UV photoexcitation of the different isomers. The lines are guides for eyes.

The antioxidant activity measured by the DPPH assay (Figure 8a) mirrors the decay time. 1,8-DHN, with its strong intramolecular H-bond, exhibits the highest radical-scavenging efficiency, reaching complete DPPH reduction at the lowest concentration.^26^ The rapid hydrogen-atom transfer^14^ and electron-transfer capacity can be ascribed to the preorganized O–H···O geometry, which lowers the bond-dissociation enthalpy and facilitates proton-coupled electron transfer. 1,5-DHN, lacking a closed intramolecular chelate but still allowing moderate intermolecular H-bonding, shows intermediate activity, whereas 1,3- and 2,7-DHN require higher concentrations to achieve comparable scavenging, consistent with the absence of intramolecular hydrogen bonding capable of stabilizing the aryloxy radical formed after H atom abstraction, despite the presence of ground-state π-delocalization. Previous studies on hydroxylated aromatics have demonstrated that intramolecular hydrogen bonding can lower the effective O–H bond dissociation energy by preferentially stabilizing the corresponding aryl-oxyl radical and therefore facilitating hydrogen-atom transfer.^27,28^ In peri-substituted systems such as 1,8-dihydroxynaphthalene, preorganized O–H···O interactions reduce the energetic barrier for radical formation and favor proton-coupled electron transfer pathways. Although no direct BDE determination has been obtained in the present work, the observed hierarchy of radical-scavenging activity is consistent with this established structure–reactivity framework.

Figure 8b shows the isocyanine green assay to measure the capability of generating singlet oxygen upon UV photoexcitation. In this case the isomers show remarkable differences. The capability of generating singlet oxygen follows the order: 1,3 DHN > 1,8 DHN > 1,5 DHN > 2,7. The indocyanine-green photobleaching assay (Figure 8b) highlights a different trend for singlet-oxygen generation. The order 1,3 > 1,8 > 1,5 > 2,7 indicates that 2,7-DHN, despite its extended conjugation, appears photochemically inert due to inefficient intersystem crossing associated with its symmetric π-framework and weak spin–orbit coupling. The partial sensitizing behavior of 1,8-DHN reflects a competition between ESIPT-mediated quenching and triplet formation: its strong H-bond stabilizes the singlet state but also introduces vibronic coupling that can open a minor ISC pathway. Thus, the isomeric series delineates a clear photophysical crossover, from antioxidant to photosensitizer, regulated by the topology of the hydroxy groups and the balance between singlet- and triplet-state deactivation routes.

Overall, the combined lifetime, DPPH, and singlet-oxygen results well support the structure–function model established from the vibrational and electronic analyses. Intramolecular hydrogen bonding drives energy dissipation and antioxidant behavior, while symmetry-preserving or weakly coupled geometries promote intersystem crossing and ROS generation. These findings provide a coherent mechanistic framework for tailoring hydroxylated aromatic systems as either photoprotective antioxidants or controlled singlet-oxygen sensitizers through rational manipulation of hydrogen-bond topology and π-electron delocalization.

### 3.6 Photophysical Model of DHN Isomers

The collective spectroscopic and reactivity data delineate a unified model in which hydrogen-bond topology and π-electron delocalization act as the two principal parameters governing the excited-state dynamics of dihydroxynaphthalenes. The interplay between these structural factors determines whether the photoexcited system relaxes through energy dissipation (antioxidant pathway) or energy transfer (photosensitizing pathway).

FTIR and UV–vis results demonstrate that the position of the hydroxy groups modulates both the degree of π-conjugation and the strength of intra- versus intermolecular hydrogen bonding. 1,8-DHN forms a strong *peri* O–H···O intramolecular hydrogen bond, generating a quasi-chelated, anisotropic π-system. 1,5-DHN supports weaker and asymmetric H-bonding, largely intermolecular. 1,3-DHN exhibits localized, resorcinol-like electron density with minimal conjugation across the naphthalene core and finally 2,7-DHN, with para-like substitution on opposite rings, displays the most extended π-delocalization but no intramolecular H-bonding.

Upon π → π* excitation, all DHNs populate a lowest singlet excited state (S_1_) derived from the ^1^L_b_ manifold. The subsequent relaxation path depends on the coupling between electronic and nuclear motions. 1,8-DHN undergoes partial excited-state intramolecular proton transfer (ESIPT) within the *peri* H-bond, producing dual emissive species with distinct lifetimes (τ_1_ ≈ 2.5 ns, τ_2_ ≈ 11.7 ns). This process enhances internal conversion and nonradiative decay. 1,3- and 1,5-DHN exhibit predominantly single-exponential decays dominated by neutral S_1_ fluorescence, despite evidence of excited-state heterogeneity in 1,3-DHN. 2,7-DHN shows the shortest lifetime (τ ≈ 5.8 ns), consistent with a rigid, symmetric π-framework that favors fast singlet-state relaxation while limiting efficient intersystem crossing and energy transfer to molecular oxygen.

The overall properties can be represented by two competing pathways after optical excitation

S0→hνS1⇒{internalconversionESIPT→heat,fluorescence(antioxidantbranch)intersystemcrossing→T1→O21generation(photosensitizerbranch)

The branching ratio between these channels is governed by the geometry of hydrogen bonds and the extent of π-delocalization: tighter intramolecular coupling (*peri* H-bonds) favors nonradiative quenching and antioxidant activity, while open, symmetric systems promote triplet formation and ROS generation.

In essence, positional isomerism in DHNs acts as a molecular trigger for energy-dissipating (1,8-DHN type) and/or energy-transferring (1,3-DHN type) regimes. This structure–property relationship provides a predictive framework for designing hydroxylated aromatics with tailored photochemical outcomes, from photoprotective antioxidants to efficient singlet-oxygen sensitizers, simply by manipulating the spatial arrangement of hydroxy groups on the aromatic scaffold.

It is worth noting that that no attempt was made to extract hydrogen-bond association energies or O–H bond dissociation enthalpies from the present data. Quantitative determination of such parameters requires solution-phase equilibrium and kinetic measurements under carefully controlled conditions, as already established in detailed studies of hydroxylated aromatics.^24,25^ The present work, instead, focuses on correlating hydroxy-group topology with excited-state dynamics and functional redox outcomes, using spectroscopy and comparative assays as complementary probes.

## 4 Conclusions

This study establishes a clear structure–property relationship among the four dihydroxynaphthalene isomers, demonstrating that the spatial arrangement of hydroxyl groups dictates both their electronic structure and photochemical reactivity. FTIR and UV–vis analyses revealed that intramolecular hydrogen bonding and π-conjugation are the key structural variables governing excited-state relaxation. The photoluminescence and lifetime results confirmed that these factors control the balance between radiative and nonradiative pathways: 1,8-DHN exhibits dual emissive lifetimes due to *peri* O–H···O coupling and excited-state intramolecular proton transfer, whereas 2,7-DHN, with its symmetric and extended π-framework, shows relatively fast excited-state decay dominated by singlet-state relaxation rather than efficient intersystem crossing.

Functional assays corroborate these photophysical trends. The DPPH test identified 1,8-DHN as the most effective radical scavenger, supported by its preorganized hydrogen-bond geometry that facilitates proton-coupled electron transfer. In contrast, the isocyanine-green assay revealed that the 1,3-DHN preferentially populate triplet states and act as efficient singlet-oxygen generators. The observed inverse correlation between relative radical-scavenging activity (under DPPH conditions) and ^1^O_2_ generation reflects a structural tuning between energy dissipation and energy transfer.

Overall, the DHN family exemplifies how hydroxy-group topology can function as a molecular trigger between photoprotective (radical scavenging) and photosensitizing (ROS-producing) regimes. This framework provides fundamental design principles for engineering hydroxylated aromatic systems with targeted photoredox functions, ranging from light-activated antioxidants to controllable singlet-oxygen sensitizers for photodynamic and photocatalytic applications.

## Supplementary Material



## Notes

Femtosecond transient absorption map of 1,3-DHN, 1,5 DHN, 1,8-DHN and 2,7-DHN up to 800 ps pump–probe delay following excitation at 266 nm with 100 fs pulses; transient absorption spectra at selected delay times and kinetic traces at selected probe wavelengths fitted with a three-exponential model (Figures S1–S4) (PDF)
