# Supplementary material for: Hydroxy-Group Topology as a Molecular Trigger Between Antioxidant and Photosensitizing Properties in Dihydroxynaphthalenes
Source: ACS Omega. 2026 May 28;11(22):32881–92. doi: 10.1021/acsomega.6c02192 (PMC13261408; doi:10.1021/acsomega.6c02192)
Supplement: Supplementary file 1 [file ao6c02192_si_001.pdf]

SUPPORTING INFORMATION

Hydroxy-Group Topology as a Molecular Trigger between Antioxidant and Photosensitizing Properties in Dihydroxynaphthalenes

Plinio Innocenzi*[a], Vasilis Petropoulos[b], Giulio Cerullo[b], Federico Olia[a], Davide Carboni[a], Luca Malfatti[a]

[a] Laboratory of Materials Science and Nanotechnology, CR-INSTM, Department of Engineering, University of Sassari. Via Vienna 2, 07100 Sassari, Italy.

[b] Department of Physics, Politecnico di Milano, Piazza Leonardo da Vinci, 32, 20133 Milan, Italy.

* Corresponding author: plinio@uniss.it

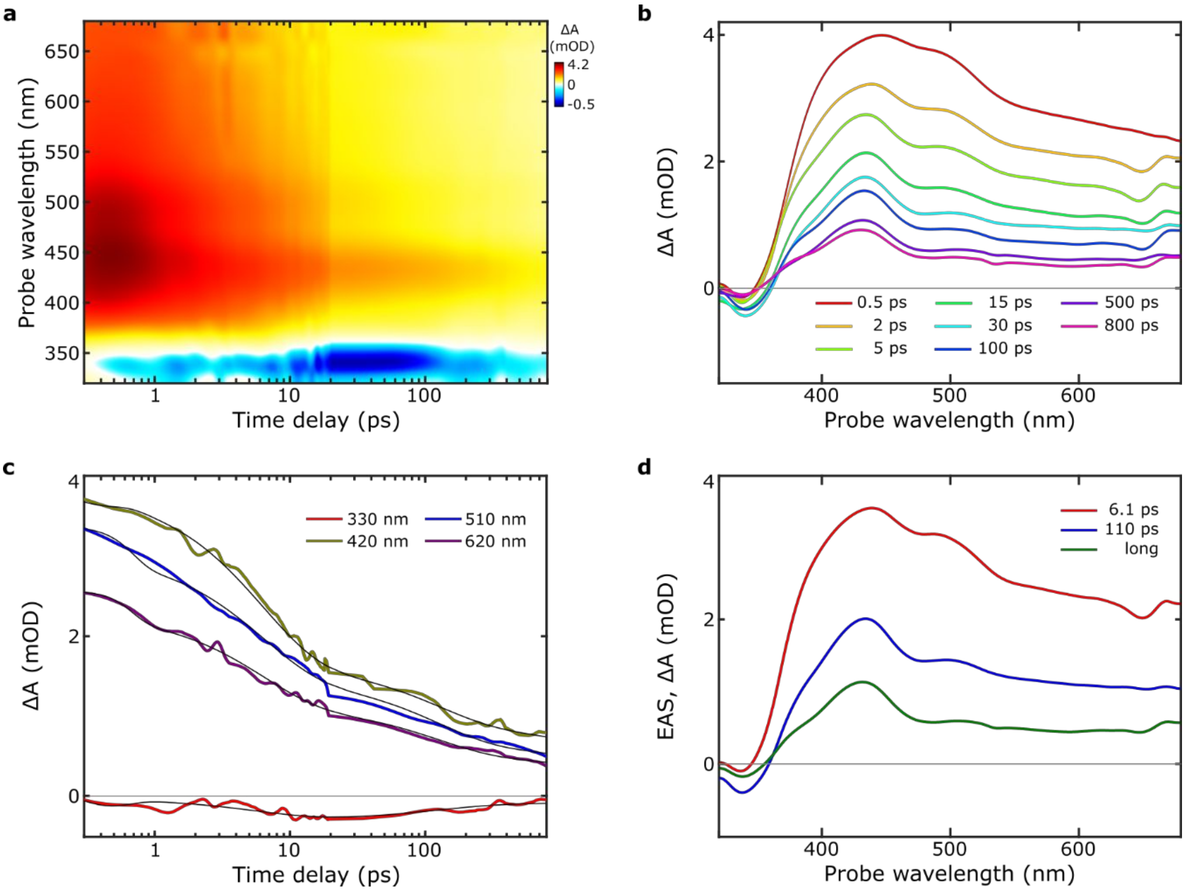

Figure S1. (a) Femtosecond transient absorption (fs-TA) map of 1,3-DHN up to 800 ps pump–probe delay following excitation at 266 nm with 100 fs pulses. (b) Transient absorption spectra at selected delay times. (c) Kinetic traces at selected probe wavelengths fitted with a three-exponential model. (d) Evolution-associated spectra (EAS) and corresponding time constants obtained from global analysis.

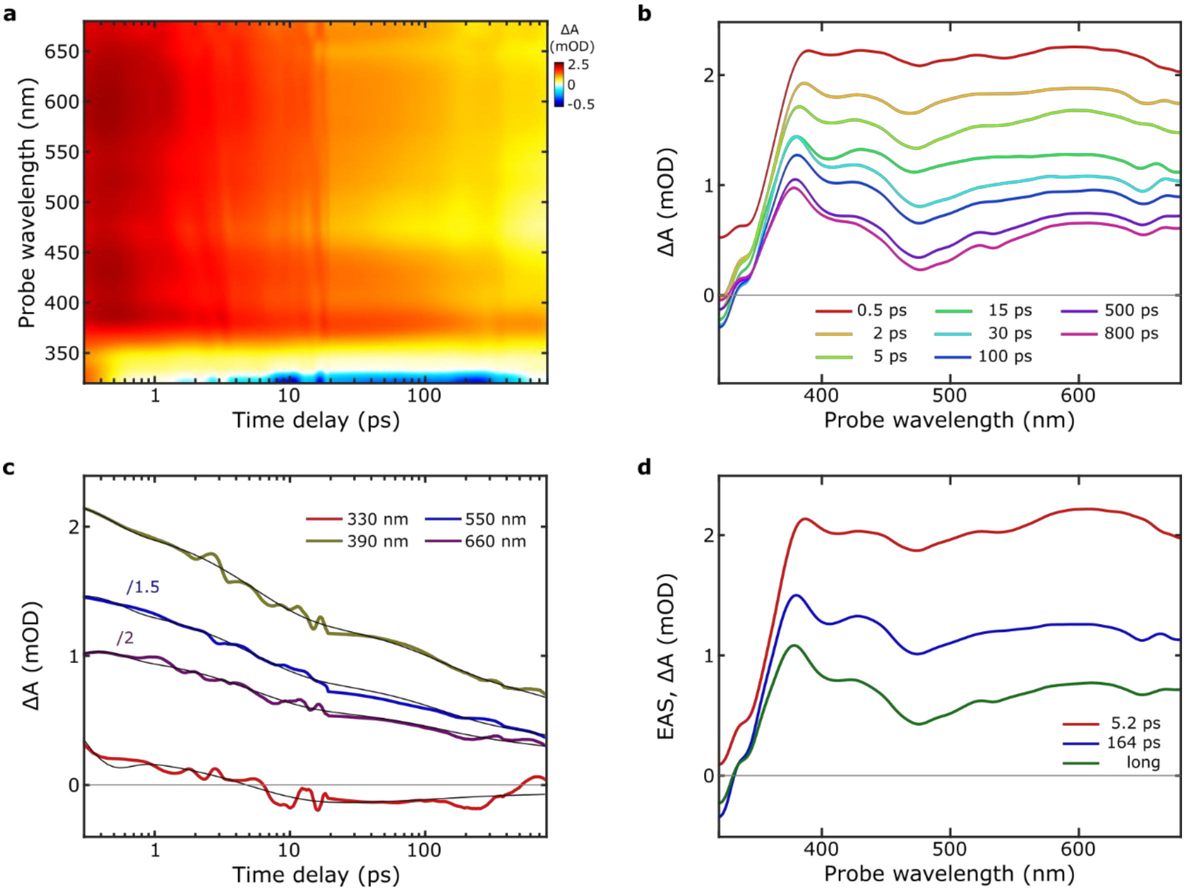

Figure S2. (a) Fs-TA map of 1,5-DHN up to 800 ps pump–probe delay following excitation at 266 nm. (b) TA spectra at selected delay times. (c) Kinetic traces at selected probe wavelengths fitted with a three-exponential model; traces recorded at 550 nm and 660 nm were scaled for clarity. (d) EAS components and corresponding time constants obtained from global analysis.

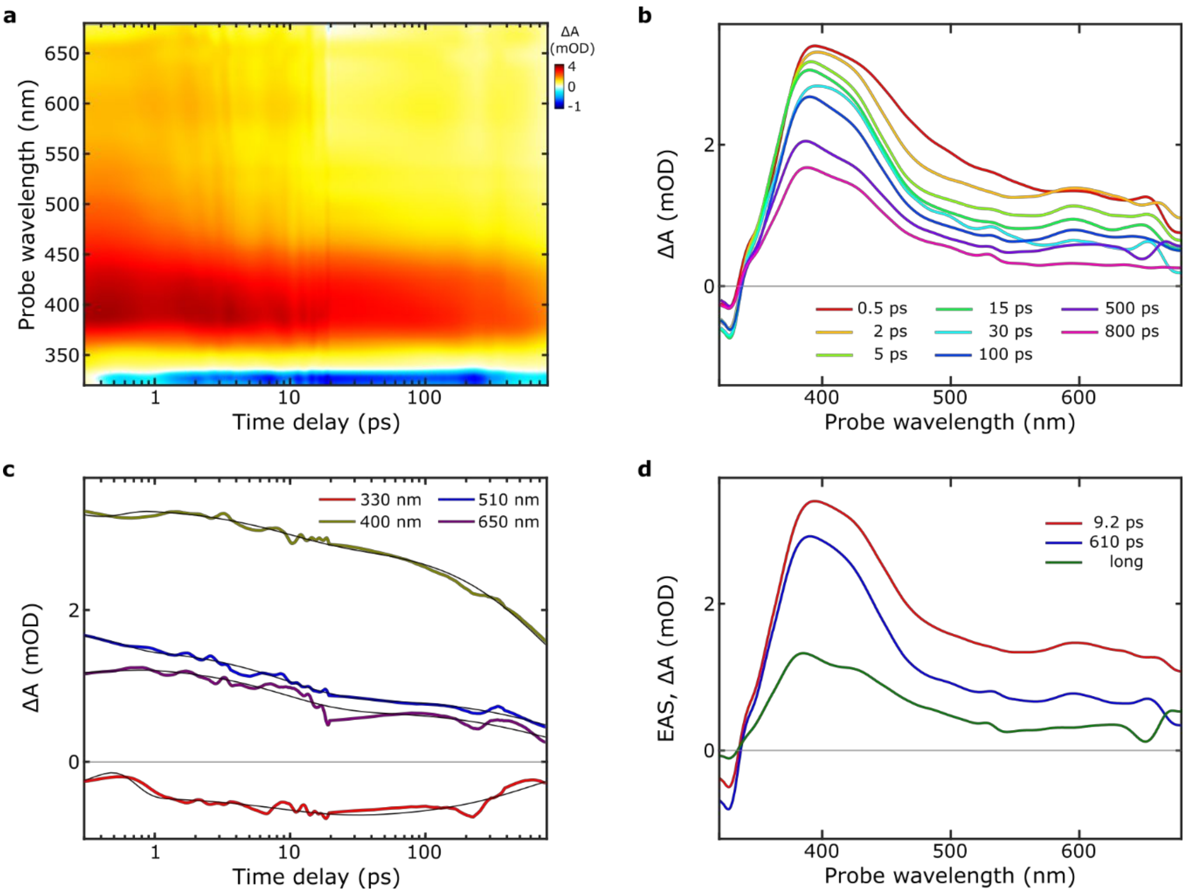

Figure S3. (a) Fs-TA map of 1,8-DHN recorded up to 800 ps pump–probe delay following excitation at 266 nm. (b) TA spectra shown at selected delay times. (c) Temporal traces at selected probe wavelengths fitted with a three-exponential model. (d) EAS components together with the corresponding time constants obtained from global analysis.

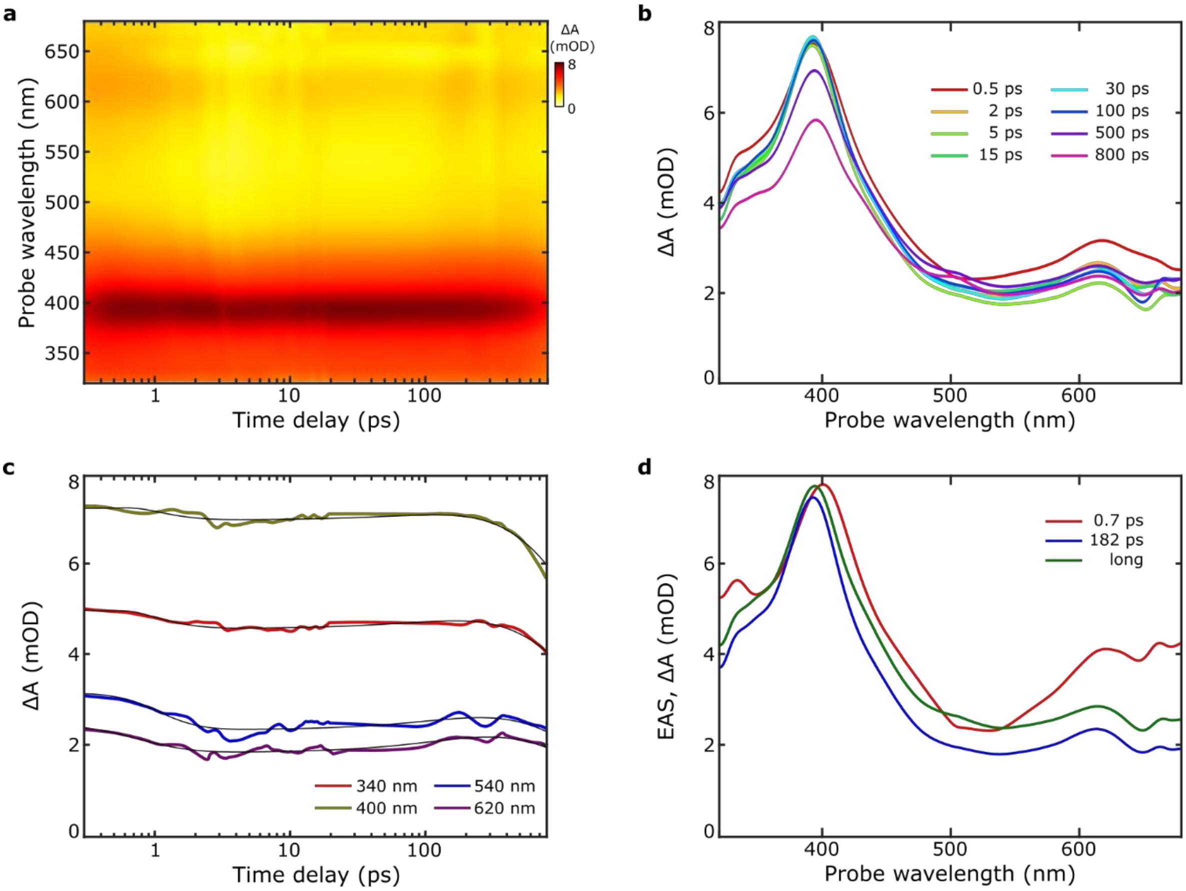

Figure S4. (a) Fs-TA map of 2,7-DHN up to 800 ps pump–probe delay following excitation at 266 nm. (b) TA spectra at selected delay times. (c) Kinetic traces at selected probe wavelengths fitted with a three-exponential model. (d) EAS components and corresponding time constants obtained from global analysis.
